# P-56 - THE IMPACT OF LONG-COVID PERSISTENT SYMPTOMS FOLLOWING SARS-COV-2 INFECTION AMONG HEALTHCARE WORKERS: CROSS-SECTIONAL FINDINGS FROM THE UK SIREN COHORT IN 2025

**DOI:** 10.1093/pubmed/fdag046.087

**Published:** 2026-07-09

**Authors:** Dr Irina Lut, Katie Munro, Quisha Bustamante, Dr Ana Atti, Dr Jasmin Islam, Jameel Khawam, Dr Colin Brown, Prof Susan Hopkins, Dr Emma Wall, Victoria Hall, Sarah Foulkes

**Affiliations:** UK Health Security Agency; UK Health Security Agency; UK Health Security Agency; UK Health Security Agency; UK Health Security Agency; UK Health Security Agency; UK Health Security Agency; UK Health Security Agency; The Francis Crick Institute; University College Hospitals NHS Foundation Trust Biomedical Research Centre; Centre for Immunobiology and Infection, Blizard Institute, Queen Mary University of London; UK Health Security Agency; UK Health Security Agency

## Abstract

**Background:**

Approximately 6-10% of adults develop Long-COVID (LC) following SARS-CoV-2 infection. Evidence about LC in healthcare workers (HCW) is limited. SIREN is a prospective cohort study of HCW from across the four nations since 2020.

**Objectives:**

We aimed to assess the impact of LC on SIREN participants and describe experiences of persistent symptoms in the context of their work and personal life.

**Methods:**

All SIREN participants who had not opted-out of receiving communications (n=29,299) were invited to complete a cross-sectional online survey in May 2025. We calculated the proportion of participants self-reporting LC, defined as symptoms lasting at least 12 weeks following a SARS-CoV-2 infection. We calculated proportions for reported symptoms, healthcare attendance, impact on work and personal life, and compared those still experiencing LC with those who had LC recovered. We used logistic regression models to identify factors associated with LC recovery.

**Results:**

Of 4,907 respondents, 1,147 self-reported LC. 52% of respondents with LC (600/1,147) reported still experiencing LC symptoms, with 11% reported currently experiencing severe fatigue. Respondents reported LC severely affected their leisure and exercise (14%), sleep quality (12%) and hobbies (10%). Respondents were more likely to recover if they were under 45 years old (OR=1.52; 95%CI:1.1-2.1). They were less likely to recover from LC if they were experiencing severe fatigue (OR=0.21; 95%CI:0.13-0.33), attended any healthcare service (OR=0.35; 95%CI:0.27-0.45), took more than 3 months off work (OR=0.34; 95%CI:0.2-0.59) and changed their working patterns (OR=0.36; 95%CI:0.28-0.46).

**Conclusions:**

Findings, subject to response bias, indicate a substantial LC burden among HCW and negative impacts on their professional and personal lives. Reports of sustained persistent symptoms due to SARS-CoV-2 infection among HCWs highlight the need for improved occupational health support and workplace policies which could contribute to LC recovery. Further research on determinants of LC recovery is needed to inform targeted intervention and/or treatment.

